# Complement receptor-3 negatively regulates the phagocytosis of degenerated myelin through tyrosine kinase Syk and cofilin

**DOI:** 10.1186/1742-2094-9-166

**Published:** 2012-07-09

**Authors:** Smadar Hadas, Maya Spira, Uwe-Karsten Hanisch, Fanny Reichert, Shlomo Rotshenker

**Affiliations:** 1 Dept. of Medical Neurobiology, IMRIC, Hebrew University Faculty of Medicine, Ein-Kerem, 12272, Jerusalem, 91120, Israel; 2 Sheba Medical Center, Ramat-Gan, Israel; 3 Institute of Neuropathology, University of Gottingen, Gottingen, Germany

**Keywords:** Microglia, Macrophage, Phagocytosis, Myelin, Complement receptor-3, Syk, Cofilin

## Abstract

**Background:**

Intact myelin, which normally surrounds axons, breaks down in Wallerian degeneration following axonal injury and during neurodegenerative diseases such as multiple sclerosis. Clearance of degenerated myelin by phagocytosis is essential since myelin impedes repair and exacerbates damage. CR3 (complement receptor-3) is a principal phagocytic receptor in myelin phagocytosis. We studied how tyrosine kinase Syk (spleen tyrosine kinase) and cofilin control phagocytosis of degenerated myelin by CR3 in microglia and macrophages. Syk is a non-receptor tyrosine kinase that CR3 recruits to convey cellular functions. Cofilin is an actin-depolymerizing protein that controls F-actin (filamentous actin) remodeling (i.e., disassembly and reassembly) by shifting between active unphosphorylated and inactive phosphorylated states.

**Results:**

Syk was continuously activated during prolonged phagocytosis. Phagocytosis increased when Syk activity and expression were reduced, suggesting that normally Syk down regulates CR3-mediated myelin phagocytosis. Levels of inactive p-cofilin (phosphorylated cofilin) decreased transiently during prolonged phagocytosis. In contrast, p-cofilin levels decreased continuously when Syk activity and expression were continuously reduced, suggesting that normally Syk advances the inactive state of cofilin. Observations also revealed inverse relationships between levels of phagocytosis and levels of inactive p-cofilin, suggesting that active unphosphorylated cofilin advances phagocytosis. Active cofilin could advance phagocytosis by promoting F-actin remodeling, which supports the production of membrane protrusions (e.g., filopodia), which, as we also revealed, are instrumental in myelin phagocytosis.

**Conclusions:**

CR3 both activates and downregulates myelin phagocytosis at the same time. Activation was previously documented. We presently demonstrate that downregulation is mediated through Syk, which advances the inactive phosphorylated state of cofilin. Self-negative control of phagocytosis by the phagocytic receptor can be useful in protecting phagocytes from excessive phagocytosis (i.e., “overeating”) during extended exposure to particles that are destined for ingestion.

## Background

Intact myelin, which normally surrounds axons, breaks down during Wallerian degeneration following traumatic injury to axons [1-3] and during neurodegenerative diseases such as multiple sclerosis [4]. Degenerated myelin thus formed impedes repair and exacerbates damage by arresting regeneration [5-7], inhibiting remyelination [8] and advancing production of membrane attack complexes [9,10]. Therefore, rapid clearance of the degenerated myelin is critical for repair of the injured nervous system. Regarding this, it is important to elucidate the mechanisms that regulate degenerated myelin phagocytosis. We focused in this study on how Syk and cofilin regulate the myelin phagocytosis that CR3 (complement receptor-3; α_M_β_2_ integrin) mediates in primary microglia and macrophages; the term "myelin" will replace "degenerated myelin" from here onward for simplicity.

The principal phagocytic receptors for myelin on macrophages and microglia are CR3, SRA (scavenger receptor-AI/II), and Fcγ [11-14]. CR3 functions both as a C3bi/opsonic and unopsonic receptor for C3bi-opsonized and unopsonized myelin, respectively. Unopsonic SRA primarily mediates phagocytosis of unopsonized myelin. However, Fcγ, but not CR3 and SRA, requires prior opsonization of myelin by anti-myelin Abs. We studied myelin phagocytosis by CR3 and SRA in the absence of anti-myelin Abs.

Syk is a non-receptor tyrosine kinase that phagocytic receptors Fcγ, CR3, and Dectin-1 recruit upon their activation [15-19]. However, Syk may or may not activate phagocytosis depending on the type of phagocyte, the identity of the phagocytic receptor, and the nature of the ingested particle [20-24]. It is important, therefore, to study each phagocyte, receptor, and phagocytosed particle combination on its own.

**Figure 1:**
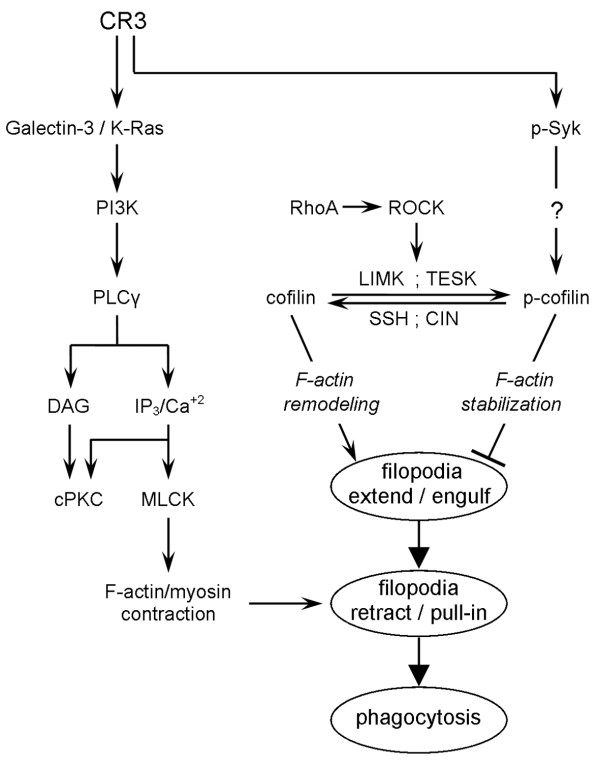
**CR3 both activates and downregulates myelin phagocytosis – a schematic representation of the working hypothesis and experimental design.** Binding of myelin to CR3 initiates structural changes characteristic of phagocytosis (marked by ellipses). Filopodia-like membrane protrusions engulf myelin as they extend and then pull myelin in as they retract. Production of filopodia depends on F-actin remodeling, which active unphosphorylated cofilin advances (↓) and inactive p-cofilin impedes (⟂) [26-28]. Therefore, phagocytosis is expected to be reduced when cofilin is inactivated by shifting the balance from cofilin to p-cofilin through activation of LIMK and/or TESK, for example, by RhoA and ROCK, and/or downregulation of phosphatases SSH and CIN. CR3 downregulates phagocytosis by activating Syk, which in turn advances the inactive phosphorylated state of cofilin. How Syk promotes cofilin inactivation is yet to be determined. CR3 activates myelin phagocytosis by activating K-Ras and its downstream effectors in a galectin-3-dependent manner [30-33]. Subsequently, retraction of filopodia is aided by contraction that Ca^+2^-calmodulin-dependent MLCK (myosin light chain kinase) activates and F-actin and non-muscle myosin carry out [34]. Not all possible interactions are shown.

Cofilin/ADF (actin depolymerizing factor) is a family of proteins that controls F-actin remodeling and thereby the production of membrane protrusions (e.g., filopodia and lamelopodia). In mice, cofilin-1 is expressed in most cells, cofilin-2 in muscle, and ADF in epithelial and nerve cells [25]. Cofilin can regulate myelin phagocytosis by controlling F-actin remodeling and filopodia production since filopodia are involved in myelin phagocytosis (Figure 1). First, filopodia engulf myelin as they extend, and then, filopodia pull myelin into phagocytes as they retract. Protrusion of membranes requires local remodeling of F-actin [26-28]. In turn, retraction of membrane protrusions is aided by contraction, which is based on interactions between actin and non-muscle myosin [29]. Active unphosphorylated cofilin advances remodeling by cutting of F-actin into G-actin monomers. Then, severed F-actin may protrude cell membranes as it reassembles and grows. The transitions of cofilin between inactive and active states in inflammatory cells are mainly through phosphorylation and dephosphorylation.

We document in this study that CR3 activated Syk, which in turn downregulated CR3-mediated phagocytosis of C3bi-opsonized and unopsonized myelin in CNS microglia and bone marrow-derived macrophages by advancing the inactive phosphorylated state of cofilin (Figure 1). Syk-dependent downregulation of phagocytosis and Syk-dependent control of cofilin activation have not been reported previously. These observations suggest a novel mechanism by which CR3 can both activate and downregulate myelin phagocytosis in parallel. This self-negative control of phagocytosis by CR3 can be useful in protecting phagocytes from excessive phagocytosis during extended exposure to degenerated myelin and possibly also to other particles that are destined for ingestion.

## Methods

### Animals

Wild-type BalbC and C57BL6 mice (Harlan, Israel), and BalbC mice lacking SRAI/II (SRAI/II^−/−^) or C57BL6 mice lacking α_M_/CD11b subunit of CR3 (CR3^−/−^) were used and handled in accordance with the National Research Council’s guide for the care and use of laboratory animals and the approval of the institutional committee.

Primary microglia were isolated from neonate mice brains [13]. In brief, brains were stripped of their meninges, enzymatically dissociated, cells plated on poly-L-lysine-coated flasks for 1 week, replated for 1 to 2 h on bacteriological plates, and non-adherent cells washed away. The vast majority of adherent cells (over 95 %) are microglia judged by morphology and positive immunoreactivity to the murine-specific monocyte, macrophage, and microglia marker F4/80, and the activation markers galectin-3/MAC-2 and CR3. Microglia were propagated by incubation in medium containing 10 % conditioned medium by the L-cell line, which produces M-CSF.

Primary peritoneal macrophages were harvested in cold DMEM/F12 3 to 4 days after intraperitoneal injection of 1.5 ml of 3 % thioglycollate (Difco, USA), plated in the presence of 10 % heat-inactivated FCS in 96-well culture plates (Nunc International, USA) for 2 h, and non-adherent cells washed away. The remaining adhered cells are macrophages since they express F4/80, galectin-3/MAC-2, and CR3 [12].

### Myelin isolation

Myelin was isolated from mice brains as previously described [35].

C3bi-opsonization was carried out by pre-incubating myelin in 50 % fresh mouse or rat serum in DMEM/F12 for 40 min at 37° C, followed by washing in serum-free media as previously described [34,36]. Levels of phagocytosis were the same whether mouse or rat sera were used, indicating similar opsonization efficiency by the two. Additionally, experiments that were performed in the presence of 10 % HI (heat inactivated)-rat serum, 10 % HI-mouse serum, 10 % HI-FCS, or 0.1 % delipidated BSA did not differ, indicating that heat inactivation of the complement system was effective in all sera. We used, therefore, mostly rat serum, which is easier to obtain in the quantities required to opsonize myelin, and HI-FCS as supplement in the phagocytosis assays. The efficiency of C3bi-opsonization was tested in each individual experiment by validating that levels of phagocytosis of C3bi-opsonized myelin are about two fold higher than those of unopsonized myelin.

### Myelin phagocytosis

Microglia or macrophages were plated in 96-well tissue culture plates at a density that minimizes cell-cell contact (2.5 × 10^4^/well) in the presence of DMEM/F12 supplemented with 10 % heat inactivated (HI) rat serum, 10 % HI mouse serum, 10 % HI-FCS, or 0.1 % delipidated BSA (Sigma-Aldrich, Israel), thus in the absence of externally provided complement. Phagocytes were left to rest overnight, washed, and myelin was added for the indicated times. Then unphagocytosed myelin was washed out and levels of phagocytosis quantified. At this time all remaining myelin has already been internalized/phagocytosed [34]. When phagocytosis was assayed in the presence of Syk inhibitors, phagocytes were pre-incubated in the presence of either SYK-inhibitor or piceatannol (Calbiochem, USA), or control/vehicle for 15 min, and phagocytosis assayed thereafter in the continued presence of inhibitor/vehicle.

ELISA assay to quantify myelin phagocytosis is based on the detection of the myelin-specific MBP (myelin basic protein) in lysates of phagocytes [35]. Since MBP is unique to PNS and CNS myelin, and is not produced by phagocytes, MBP levels detected in cytoplasm are proportional to levels of phagocytosed myelin. In brief, after non-phagocytosed myelin is washed away, phagocytes are lysed, lysates transferred to high-protein absorbance plates (Nalge Nunc International, USA), and levels of MBP determined by ELISA using rat anti-MBP (Serotec, Oxford, UK). We determined previously that more than 95 % of the detected MBP arises from phagocytosed/internalized myelin [35]. We further verified the validity of this phagocytosis assay by testing the ability to detect inhibition of myelin phagocytosis by cytochalasin-D [36].

### Generation of microglia with stable reduced Syk and cofilin expression

Reduction of Syk expression was achieved through lentiviral infection of wild-type BalbC microglia with short hairpin RNAs directed against mouse Syk mRNA using pLKO.1 puro plasmids (Sigma-Aldrich, Israel). The shRNA sequence selected in the Syk cDNA coding sequence was 5'CGGCGAAGGGAAAGTATTGCACTACTCGAGTAGTGCAATACTTTCCCTTCGTTT3'. The plasmid was transfected into a 293 T-based packaging cell line, and the resulting culture supernatant was used for lentiviral infection of microglia. Infected microglia were selected on the basis of their resistance to puromycin (Sigma-Aldrich, Israel) brought by the pLKO.1 plasmid, and their level of Syk protein expression was monitored by immunoblotting. As control, microglia were infected in a similar way with the shRNA sequence 5'CTTACGCTGAGTACTTCGA-3' against the non-target firefly Luciferase gene (a gift from Dr. I. Ben-Porath).

### Immunoblotting

Phagocytes were washed in PBS and lysed by incubation in ice-cold lysis buffer (Tris-HCL 1 M, pH 7.5, MgCl2 1 M, NaCl 4 M, 0.5 % NP-40, 0.1 % DTT, and 0.1 % NaVa) supplemented with protease and phosphatase inhibitor cocktail (Sigma-Aldrich, Israel), cellular debris was removed by centrifugation, and total protein determined using Bradford reagent (Sigma-Aldrich, Israel). Equal protein content lysates were separated on 10 % SDS-PAGE to detect Syk and 12 % SDS-PAGE to detect cofilin. Proteins were blotted to nitrocellulose membranes, blocked with 10 % non-fat milk or 5 % BSA in TBS (Tris-buffered saline) for 1 h at RT, incubated over night at 4° C in the presence of primary Abs rabbit anti-cofilin, rabbit anti-p-cofilin-1 and rabbit anti-Syk (Santa Cruz Biotechnology, USA), rabbit anti-p-Syk (Cell Signaling, USA), and mouse anti-actin mAb (MP Biomedicals, USA). Blots were washed with TBST, and incubated with respective secondary Abs goat anti-rabbit and goat anti-mouse conjugated to HRP (Jackson ImmunoReserach, USA) for 40 min at RT. Proteins were visualized with EZ-ECL kit for HRP detection (Beit Haemek, Israel). The intensities of immunoblot bands were determined by TINA software, and quantification was carried out as indicated in the figure legends.

### Confocal fluorescence microscopy and time-lapse cinematography

Myelin was pre-labeled by DiIc18 (Molecular Probes). Then 1 μl of DiIc18 stock solution (5 mM in DMSO) was added to 1 ml of myelin for 15 min at 37° C; myelin washed (× 3 in culture medium) and then added to macrophages. Macrophage plasma membrane was labeled by dye RH237 (N-(4-sulfutyl)-4-(6-(p-dibutylamynophenyl) hexatrenyl) pyridinium, inner salt (Molecular Probes). A stock solution of 10-mM RH237 in ethanol was diluted before use in culture medium to a final concentration of 0.5 μM. Images were taken in the continuous presence of the dye. Therefore, some of the myelin, which was pre-labeled by DiIc18, acquired some of the RH237. Ca^+2^ sensor Fluo-4 (Molecular Probes) was used to image the cytoplasm of macrophages by imaging intracellular Ca^+2^. Macrophages were incubated in Fluo-4/AM solution (50 μg Fluo-4/AM dissolved in 10 μl DMSO to which 500 μl culture medium and 10 μl of 1 μg/μl pluronic acid were added) for 40 min and then washed (× 3 in culture medium).

The system used for confocal imaging consisted of an Olympus microscope IX70 and a Bio-Rad Radiance 2000/AGR-3 confocal imaging system. RH237 was excited at 514 nm (argon laser) and emitted fluorescence collected by a 660-nm low-pass filter. DiIc18 was excited at 543 nm (green HeNe laser) and emitted fluorescence collected at 555–625 nm. Fluo-4 was excited at 488 nm (argon laser) and emitted fluorescence collected at 500-530 nm. The argon laser excitation intensity was usually lowered to 5-10 %. The pinhole was set to 1.6 to 2.5 mm. A single optical slice was continuously but sequentially scanned for DiIc18, RH237, and Fluo-4 (each lasting 9 s). Images were collected and processed using LaserSharp, LaserPix, and LaserVox (BioRad software).

### Media products

Media products DMEM, DMEM/F12, FCS, HI-FCS, gentamycin sulfate, and L-glutamine were obtained from Biological Industries (Beit-Haemek, Israel).

### Statistical analysis

Mann-Whitney and one- and two-way ANOVA analyses were carried out as indicated in the figure legends.

## Results

### Syk is activated during phagocytosis of degenerated myelin

**Figure 2:**
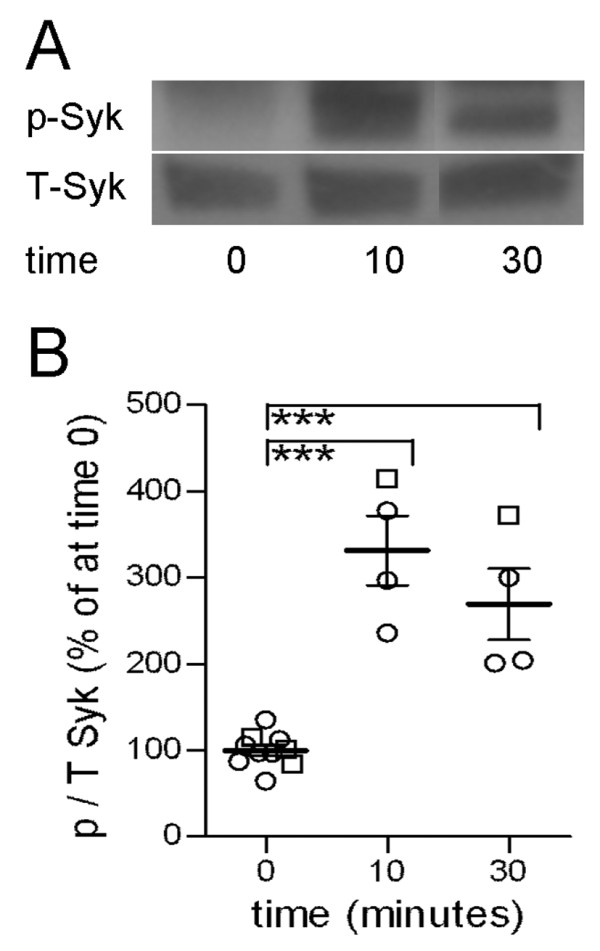
**Syk is phosphorylated during phagocytosis of C3bi-opsonized myelin.** (**A**) Total (T) and phospho (p) Syk levels were determined by immunoblot analysis in lysates of wild-type and control Con-Luc microglia before (0 min), and after 10 and 30 min of phagocytosis. (**B**) The ratio p/T at 10 and 30 min was calculated for each microglia type as percentage of p/T in respective non-phagocytosing phagocytes (i.e., at 0 min), which was defined as 100 %. Averages ± SEM of individual experiments, each performed in triplicates, are given. Significance of difference by one-way ANOVA and the Dunnet post test is *** *p* < 0.001. The immunoblot in (**A**) is from control Con-Luc microglia. Data in (**B**) were obtained from wild-type (squares) and Con-Luc (circles) microglia.

Phagocytosis of myelin in the absence of anti-myelin Abs is mediated jointly by CR3 and SRA, albeit two to four fold more by CR3 than SRA [13]. Taken that Syk is activated by phosphorylation during CR3-mediated Syk-depended functions [15,16], we tested whether Syk is also phosphorylated during myelin phagocytosis in wild-type BalbC microglia and microglia infected with the non-target luciferase-shRNA that were used in subsequent experiments as control (Con-Luc) for microglia that were infected with Syk-shRNA. Levels of p-Syk (phosphorylated Syk) increased substantially for the entire 30-min period of phagocytosis tested, indicating that Syk was continuously activated during prolonged phagocytosis (Figure 2).

### Syk down-regulates CR3-mediated myelin phagocytosis

**Figure 3:**
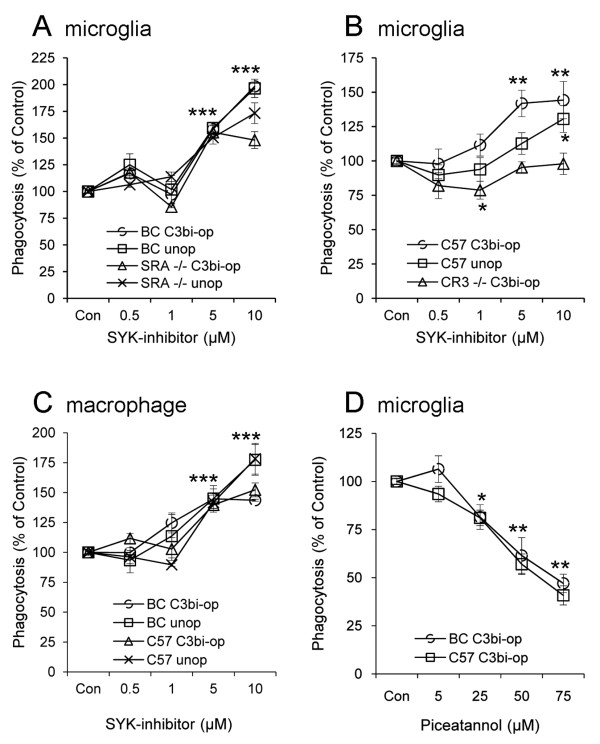
**SYK-inhibitor augments CR3-mediated phagocytosis of C3bi-opsonized and unopsonized myelin in microglia and macrophages.** SYK inhibitor augmented phagocytosis of C3bi-opsonized (C3bi-op) and unopsonized (unop) myelin in (**A**) wild-type BalbC (BC) and SRA^−/−^ microglia, (**B**) wild-type C57BL (C57) but not CR3^−/−^ microglia, and (**C**) wild-types BalbC and C57BL macrophages. (**D**) Piceatannol inhibited phagocytosis of C3bi-opsonized myelin by wild-type BalbC and C57BL microglia. Levels of phagocytosis that were determined after 30 and 60 min of exposure to myelin were similar and thus combined. Phagocytosis of C3bi-opsonized and unopsonized myelin in control/vehicle (Con) was each defined as 100 %. Phagocytosis at indicated concentrations of either SYK inhibitor or piceatannol was calculated as percentage of respective control. Averages ± SEM of six to ten experiments, each performed in triplicates, and levels of significance by one-way ANOVA and the Dunnett post test are: * *p* < 0.05, ** *p* < 0.01, *** *p* < 0.001. In (**A**) and (**C**), *p* < 0.001 for all values at 5 μM and 10 μM. In (**D**), *p* < 0.05 for all values at 25 μM and *p* < 0.01 for all values at 50 μM and 75 μM.

We examined next how Syk regulates myelin phagocytosis by analyzing how two distinct inhibitors of Syk affected phagocytosis. The inhibitor “SYK inhibitor" [37] augmented phagocytosis of C3bi-opsonized and unopsonized myelin, which is mediated jointly by CR3 and SRA in wild-type BalbC microglia and by CR3 without SRA in BalbC SRA^−/−^ microglia (Figure 3A). SYK inhibitor augmented phagocytosis of C3bi-opsonized and unopsonized myelin by CR3 and SRA combined in wild-type C57BL microglia but produced inhibition of phagocytosis by SRA without CR3 in C57BL CR3^−/−^ microglia only at 1 μM (Figure 3B). Finally, SYK inhibitor augmented phagocytosis of C3bi-opsonized and unopsonized myelin in wild-type BalbC and C57BL macrophages (Figure 3C). Taken together, SYK-inhibitor augmented phagocytosis of C3bi-opsonized and unopsonized myelin by CR3, but not by SRA. In contrast to augmentation by SYK inhibitor, phagocytosis of C3bi-opsonized myelin was reduced in wild-type BalbC and C57BL microglia by piceatannol, which was frequently used by others (e.g., [38-40], as an inhibitor of Syk (Figure 3D).

**Figure 4:**
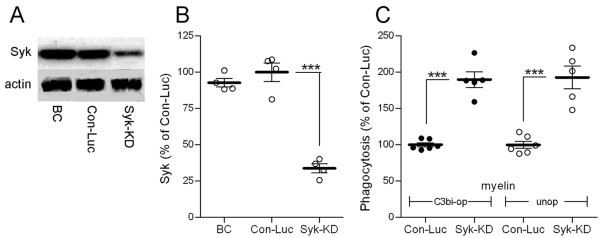
**Phagocytosis of C3bi-opsonized and unopsonized myelin is augmented in Syk knocked-down (Syk-KD) microglia.** (**A**) Immunoblot of Syk and actin in wild-type BalbC (BC), control (Con-Luc), and Syk-KD microglia. (**B**) Quantitation of Syk levels based on (A). Syk/actin ratio in BalbC and Syk-KD microglia was calculated as percentage of Syk/actin in control Con-Luc microglia, which was defined as 100 %. Values of individual experiments, averages ± SEM, and significance of differences by double-tailed Mann-Whitney is *** *p* < 0.001. (**C**) Phagocytosis of C3bi-opsonized (C3bi-op) and unopsonized (unop) myelin by Syk-KD and control Con-Luc microglia. Levels of phagocytosis that were determined after 30 and 45 min of exposure to myelin were similar and thus combined. Phagocytosis by Syk-KD microglia was calculated as percentage of phagocytosis by control Con-Luc microglia, which was defined as 100 %. Values of individual experiments, each performed in triplicates, averages ± SEM, and significance of differences by double-tailed Mann-Whitney is *** *p* < 0.001.

The opposing effects that the two inhibitors of Syk produced did not allow us to determine whether Syk activated or downregulated myelin phagocytosis. We addressed this issue further by examining how phagocytosis is affected after reducing Syk expression in phagocytes. Syk was knocked down (Syk-KD) by lentiviral infection with Syk-shRNA in wild-type BalbC microglia (Figure 4A and B). Consequently, phagocytosis of C3bi-opsonized and unopsonized myelin was augmented (Figure 4C), suggesting that normally Syk downregulates this phagocytosis. Thus, augmentation in Syk-KD microglia corresponded to augmentation by SYK inhibitor and not to inhibition by piceatannol, indicating that SYK inhibitor and not piceatannol is Syk-specific in our assay system. Therefore, SYK inhibitor and not piceatannol was used to inhibit Syk activity pharmacologically in subsequent experiments. Of note, also others questioned the specificity of piceatannol towards Syk in some cell types; see Discussion and [38-40].

### Filopodia like membrane protrusions are involved in myelin phagocytosis

**Figure 5:**
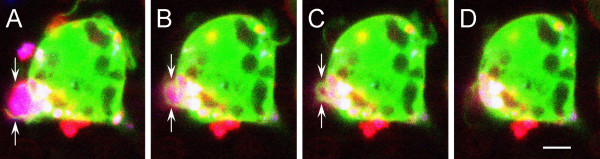
**Filopodia engulf and then pull myelin into phagocyte.** Successive steps during phagocytosis of a myelin particle by a wild-type BalbC macrophage. (**A**) Arrows mark a filopodium-like structure (green and red) that engulfs a myelin particle (purple). Then (**B to D**), the filopodium retracts, pulling the myelin into the phagocyte. Phagocytosis was monitored continuously by confocal fluorescence microscopy at a single plane of focus. The macrophage cytoplasm was visualized by Ca^+2^ indicator Fura-4 (green), macrophage cell membrane by RH237 (red), and myelin by DiIc18 (blue). However, RH237 that was initially localized to the macrophage plasma membrane diffused out and became incorporated into myelin that turned purple (blue + red). Bar: 5 μm.

Syk could downregulate CR3-mediated myelin phagocytosis by affecting elements of cytoskeleton that control phagocytosis. Since phagocytosis may require engulfment of myelin by membrane protrusions, Syk could downregulate phagocytosis by impeding cofilin-dependent production of membrane protrusions. We addressed this issue by examining first if membrane protrusions are at all involved in myelin phagocytosis. Phagocytosis was monitored live by fluorescence confocal microscopy. Phagocytes produced filopodia-like membrane protrusions that engulfed myelin at initial stages of phagocytosis (Figure 5A). Then, filopodia pulled myelin into phagocytes as they retracted (Figure 5B, C and D).

### Syk down-regulates cofilin activation by advancing the inactive phosphorylated state of cofilin during myelin phagocytosis

**Figure 6:**
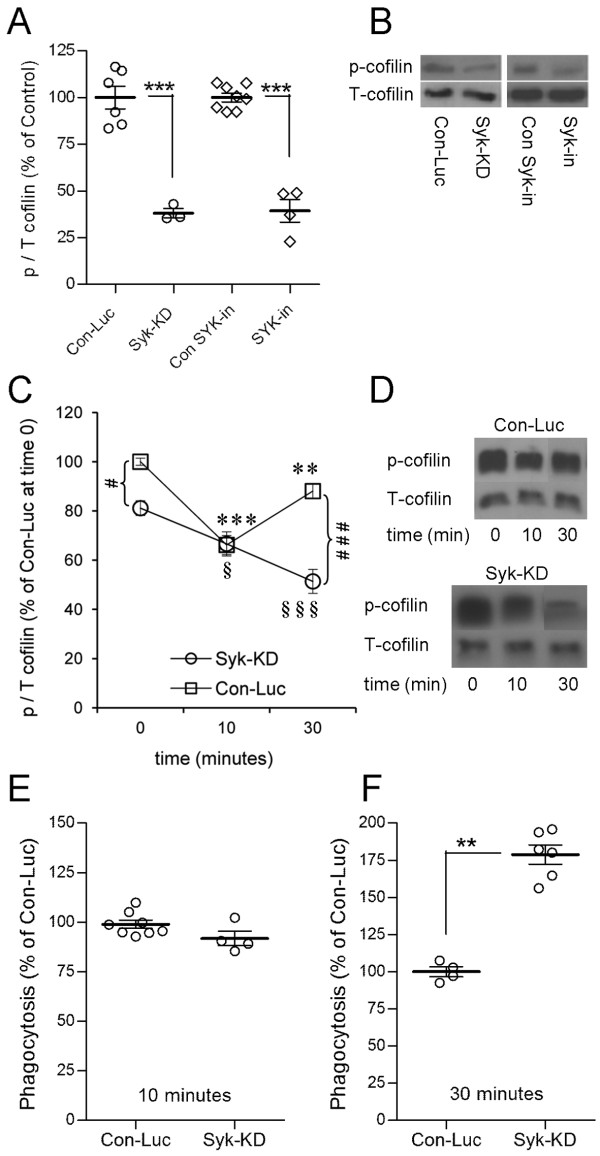
**Cofilin is continuously activated during prolonged phagocytosis when Syk is either inhibited or its expression reduced, but transiently activated during normal phagocytosis.** (**A**) Quantitation of phospho-cofilin-1 (p-cofilin) and total (T) cofilin after 45 min of phagocytosis by Syk-KD and Syk-inhibited (Syk-in) microglia and their respective controls (Con-Luc and Con SYK-in) based on (**B**) immunoblot analysis. In (A), p/T ratio in Syk-KD and Syk-inhibited microglia was calculated as percentage of p/T in their respective controls that were defined as 100 % each. Values of individual experiments, averages ± SEM, and significance of difference by double-tailed Mann-Whitney is ***p < 0.001. (**C**) Quantitation of p/T before (0 min) and after 10 and 30 min (min) of phagocytosis based on (**D**) immunoblot analysis. In (C), p/T in non-phagocytosing Con-Luc microglia was defined as 100 %. Then p/T in all other non-phagocytosing and phagocytosing microglia was calculated as percentage of p/T in non-phagocytosing control Con-Luc microglia. Average values ± SEM of three to five experiments, each performed in duplicate or triplicate, are given. Significance of differences between initial values at 0 min and those at 10 and 30 min by one-way ANOVA and the Dunnet’s post test are **p < 0.01 and ***p < 0.001 for Con-Luc microglia, and ^**§**^*p* < 0.05 and ^**§§§**^*p* < 0.001 for Syk-KD microglia. Significance of difference between Con-Luc and Syk-KD microglia by two-way ANOVA and the Bonferroni post test are ^#^*p* < 0.05 at 0 min and ^###^*p* < 0.001 at 30 min. (**E and F**) Phagocytosis by Syk-KD and control Con-Luc microglia after (**E**) 10 min and (**F**) 30 min of phagocytosis. Values of individual experiments, each performed in triplicate, averages ± SEM, and levels of significance by double-tailed Mann-Whitney are given; ** *p* < 0.01.

Active unphosphorylated cofilin promotes whereas inactive p-cofilin impedes protrusion of membranes [26-28]. Since filopodia-like membrane protrusions engulfed myelin at initial stages of phagocytosis (Figure 5), a potential mechanism through which Syk could downregulate myelin phagocytosis is by advancing the inactive phosphorylated state of cofilin. If this is the case, then Syk-KD and Syk-inhibited microglia that phagocytose more are expected to display lower levels of inactive p-cofilin (see Figure 1). Indeed, this was the case after 45 min of phagocytosis (Figure 6A and B). Further, initial levels of p-cofilin were about 20 % lower in non-phagocytosing Syk-KD microglia than in non-phagocytosing control microglia (Figure 6C and D). Then, after 10 min of phagocytosis, p-cofilin levels were reduced down to about 65 % of initial control levels in both Syk-KD and control microglia. However, as phagocytosis continued and reached 30 min, p-cofilin levels decreased further down to about 50 % of initial levels in Syk-KD microglia but returned towards initial levels in control microglia. Therefore, prolonged phagocytosis by control microglia was associated with a transient decrease in inactive p-cofilin, thus transient activation of cofilin. In contrast, augmented prolonged phagocytosis by Syk-KD and Syk-inhibited microglia was associated with a continuous decrease in inactive p-cofilin, thus continuous activation of cofilin. Further, levels of phagocytosis and levels of p-cofilin displayed inverse relationships in Syk-KD and Syk-inhibited phagocytes.

We predicted and then tested the following based on the inverse relationships between levels of phagocytosis and levels of p-cofilin. First, Syk-KD and control microglia will phagocytose about the same after 10 min since the two displayed similar levels of p-cofilin at that time. Second, Syk-KD microglia will phagocytose more than control microglia after 30 min since Syk-KD microglia displayed substantially lower levels of p-cofilin than control microglia at that time. Indeed, these predictions were confirmed as Syk-KD and control microglia phagocytosed about the same after 10 min, whereas Syk-KD microglia phagocytosed considerably more than control microglia after 30 min (Figure 6E and F).

## Discussion

We document in this study that CR3 downregulates the phagocytosis of C3bi-opsonized and unopsonized myelin in CNS microglia and bone marrow-derived macrophages through Syk, which advances the inactive phosphorylated state of cofilin. We previously documented that CR3 activates this phagocytosis through K-Ras and its downstream effector molecules in a galectin-3-dependent manner. Taken together, CR3 can both activate and downregulate myelin phagocytosis at the same time (Figure 1).

### Syk down-regulates CR3-mediated phagocytosis of C3bi-opsonized and unopsonized myelin

Syk-dependent downregulation of CR3- but not SRA-mediated phagocytosis of C3bi-opsonized and unopsonized myelin is suggested by the following observations. First, Syk was continuously phosphorylated, thus continuously activated, during prolonged phagocytosis. Then, SYK inhibitor augmented phagocytosis in SRA^−/−^ microglia where phagocytosis is CR3-dependent and SRA-independent, but not in CR3^−/−^ microglia, in which phagocytosis is SRA-dependent and CR3-independent. In accord, SYK inhibitor augmented myelin phagocytosis, which is mediated by CR3 and SRA combined in wild-type BalbC and C57BL microglia and macrophages, and in Syk-KD microglia that express reduced Syk levels.

Previous studies documented that Syk either activated or did not regulate phagocytosis at all depending on the type of phagocyte, the identity of the phagocytic receptor, and the nature of the ingested particle. Syk activated FcγR-mediated phagocytosis of IgG-opsonized erythrocytes in macrophages [20,21], and Syk activated CR3-mediated phagocytosis of C3bi-opsonized zymosan in transformed HL60 cells [22], but not in primary macrophages [21]; finally, Syk activated Dectin-1-mediated phagocytosis of unopsonized zymosan in NIH 3T3 fibroblasts transfected with Dectin-1 [24], but neither in primary macrophages nor the macrophage cell line RAW164.7 [23,24]. We presently add the novel category of Syk-dependent downregulation phagocytosis, namely the phagocytosis of C3bi-opsonized and unopsonized myelin by CR3 in primary microglia and macrophages. Our present findings suggest further that the occurrence of Syk-dependent downregulation of myelin phagocytosis is also determined by the identity of the phagocytic receptor since SYK-inhibitor augmented myelin phagocytosis by CR3 and not by SRA.

The two pharmacological inhibitors of Syk that we used produced contrasting effects, SYK-inhibitor augmented whereas piceatannol inhibited myelin phagocytosis. Of the two, SYK inhibitor (IC_50_ value of 15nM) [37] is Syk-specific in our assay system since knocking down Syk expression augmented myelin phagocytosis, as did SYK inhibitor. Inhibition by piceatannol can be explained on two grounds. First, our previous observations showed that PKA (protein kinase-A), PKC (protein kinase-C), and MLCK (myosin light chain kinase) activated myelin phagocytosis (Figure 1 and [30-33]). Second, piceatannol inhibits Syk, PKA, PKC, and MLCK with extremely close and also overlapping IC_50_ values (10, 3, 8, and 12 μM, respectively; Calbiochem). Therefore, it is very likely that piceatannol reduced myelin phagocytosis by inhibiting PKA, PKC, and MLCK alone or combined. Of note, also others have questioned the specificity of piceatannol towards Syk in some cell types [38-40].

### Cofilin regulates myelin phagocytosis

Cofilin-dependent regulation of myelin phagocytosis is suggested by the findings that levels of phagocytosis correlated positively with shifting the balance from inactive p-cofilin to active cofilin (Figure 1). First, control microglia displayed transient reduction in p-cofilin levels during prolonged phagocytosis. Second, microglia in which Syk was either inhibited or its expression reduced displayed augmentation of prolonged phagocytosis that was associated with a continuous decrease in p-cofilin. Third, control and Syk-KD microglia phagocytosed about the same at times when both displayed similar levels of p-cofilin.

Cofilin could regulate myelin phagocytosis through its ability to control remodeling of F-actin and thereby production of membrane protrusions [26-28]. This proposition is supported by our present and previous observations. Live imaging in this study revealed the involvement of filopodia-like membrane protrusions in myelin phagocytosis. Additionally, we demonstrated previously that remodeling of F-actin localized preferentially to sites of myelin phagocytosis, and, furthermore, that RhoA and its effector ROCK downregulated CR3-mediated myelin phagocytosis by stabilizing F-actin [34] (see also Figure 1).

### Syk down-regulates CR3-mediated myelin phagocytosis by advancing the inactive phosphorylated state of cofilin

Syk-dependent inactivation of cofilin is suggested by current observations that p-cofilin levels decreased when Syk activity and Syk expression were reduced. It is unclear, and yet to be determined, how Syk advanced the inactive phosphorylated state of cofilin as it could promote phosphorylation and/or impede dephosphorylation (Figure 1). To the best of our knowledge, Syk-dependent regulation of cofilin activation has not been previously reported.

Finally, the concomitant occurrences of Syk-dependent downregulation of CR3-mediated myelin phagocytosis, Syk-dependent increase in p-cofilin, and the negative correlation between phagocytosis and p-cofilin levels all together suggest that Syk downregulated phagocytosis by advancing the inactive phosphorylated state of cofilin.

### CR3 both activates and down-regulates myelin phagocytosis

All together, our present and previous [30-33] observations reveal that CR3 both activated and downregulated myelin phagocytosis, indicating that phagocytosis is controlled by the balance between the two (see also Figure 1). The dominance of activation over downregulation was accomplished, at least in part, by a transient decrease in p-cofilin levels. This transient decrease in p-cofilin was Syk-dependent since reducing Syk activity and expression resulted in a sustained decrease in p-cofilin levels that was further associated with an increase in phagocytosis.

## Conclusions

Self-negative regulation of phagocytosis by the phagocytic receptor can be useful in protecting phagocytes from excessive phagocytosis (i.e., “overeating”) during extended exposure to particles that are destined for ingestion. In our study, the phagocytic receptor was CR3 and the ingested particles the tissue debris of degenerated myelin.

## Abbreviations

ADF, actin depolymerizing factor; CIN, chronophin; CNS, central nervous system; Con-Luc, control luciferase; CR3, complement receptor-3; FcγR, Fcγ receptor; HI, heat inactivated; KD, knockdown; MBP, myelin basic protein; LIMK, LIM kinase; MLCK, myosin light chain kinase; PIP2, phosphatidylinositol (4,5)-bisphosphate; PKA, protein kinase A; PKC, protein kinase C; PLCγ, phospholipase Cγ; PNS, peripheral nervous system; sh-RNA, short hairpin RNA; SRA, scavenger receptor AI/II; SSH, slingshot homology; Syk, spleen tyrosine kinase; TESK, testis-specific kinase.

## Competing interests

The authors declare that they have no competing interests.

## Authors’ contribution

SH performed most experiments, MS performed live imaging of myelin phagocytosis, U-KH was involved in initiating the project, FR participated in most experiments, SR is heading the research group, and initiated and designed the project. All authors read and approved the final manuscript.
